# Phosphorylation of Threonine 794 on Tie1 by Rac1/PAK1 Reveals a Novel Angiogenesis Regulatory Pathway

**DOI:** 10.1371/journal.pone.0139614

**Published:** 2015-10-05

**Authors:** Jessica L. Reinardy, Daniel M. Corey, Christelle Golzio, Sarah B. Mueller, Nicholas Katsanis, Christopher D. Kontos

**Affiliations:** 1 Department of Pharmacology and Cancer Biology, Duke University Medical Center, Durham, North Carolina, United States of America; 2 Department of Medicine, Division of Cardiology, Duke University Medical Center, Durham, North Carolina, United States of America; 3 Center for Human Disease Modeling, Department of Pediatrics, Duke University Medical Center, Durham, North Carolina, United States of America; 4 Duke University School of Medicine, Duke University Medical Center, Durham, North Carolina, United States of America; Medical College of Wisconsin, UNITED STATES

## Abstract

The endothelial receptor tyrosine kinase (RTK) Tie1 was discovered over 20 years ago, yet its precise function and mode of action remain enigmatic. To shed light on Tie1’s role in endothelial cell biology, we investigated a potential threonine phosphorylation site within the juxtamembrane domain of Tie1. Expression of a non-phosphorylatable mutant of this site (T794A) in zebrafish (*Danio rerio*) significantly disrupted vascular development, resulting in fish with stunted and poorly branched intersomitic vessels. Similarly, T794A-expressing human umbilical vein endothelial cells formed significantly shorter tubes with fewer branches in three-dimensional Matrigel cultures. However, mutation of T794 did not alter Tie1 or Tie2 tyrosine phosphorylation or downstream signaling in any detectable way, suggesting that T794 phosphorylation may regulate a Tie1 function independent of its RTK properties. Although T794 is within a consensus Akt phosphorylation site, we were unable to identify a physiological activator of Akt that could induce T794 phosphorylation, suggesting that Akt is not the physiological Tie1-T794 kinase. However, the small GTPase Ras-related C3 botulinum toxin substrate 1 (Rac1), which is required for angiogenesis and capillary morphogenesis, was found to associate with phospho-T794 but not the non-phosphorylatable T794A mutant. Pharmacological activation of Rac1 induced downstream activation of p21-activated kinase (PAK1) and T794 phosphorylation *in vitro*, and inhibition of PAK1 abrogated T794 phosphorylation. Our results provide the first demonstration of a signaling pathway mediated by Tie1 in endothelial cells, and they suggest that a novel feedback loop involving Rac1/PAK1 mediated phosphorylation of Tie1 on T794 is required for proper angiogenesis.

## Introduction

Development and maintenance of the mature vasculature requires a delicate balance between vessel growth (angiogenesis) and quiescence regulated by constant integration of signals from the bloodstream and surrounding tissues [1–3]. The Tyrosine kinases with Immunoglobulin and Epidermal growth factor homology domains (Tie) receptors, Tie1 and Tie2, are known to play important roles in vascular growth, remodeling, and maintenance, as knockout of either Tie1 or Tie2 results in embryonic lethality due to vascular defects. Tie2 knockout mice die midway through gestation by embryonic day (E) 10.5 with vessel sprouting defects, whereas mice lacking Tie1 die between E13.5–15.5 or perinatally with severe vascular integrity defects, including hemorrhage and edema [4, 5].

While Tie2 and its ligands, the angiopoietins, have been fairly well characterized for their effects on vascular morphogenesis, Tie1’s role in the developing and adult vasculature has been difficult to characterize, in part because it does not directly bind any identified ligand and has weak endogenous kinase activity, making investigation of its signaling pathways difficult [6]. Using FRET analysis, Tie1 and Tie2 have been shown to interact in a ligand-independent manner [7], and Tie1 appears to play a role in limiting Tie2 signaling, as evidenced by increased Tie2 tyrosine phosphorylation following Tie1 knockdown [8]. However, Tie1’s overall function and mechanisms of action remain poorly understood.

As a receptor tyrosine kinase (RTK), Tie1 contains a single transmembrane domain and a canonical cytoplasmic kinase domain. The Tie1 protein has a high degree of homology with Tie2, with 75% amino acid identity in their kinase domains [9, 10]. Interestingly, the ~50 amino acid juxtamembrane (JM) regions of Tie1 and Tie2 are highly divergent, with the JM region of Tie1 containing 10 serine/threonine residues that are not conserved in Tie2. Although not well described, serine/threonine phosphorylation has been shown to play a role in the regulation of other RTKs, including the IGF, EGF, PDGF, and FGF receptors [11–15]. Thus, we speculated that these residues in Tie1 could serve as sites of phosphorylation and subsequent modulation of Tie1 activity and/or function, which could partially account for functional differences between Tie1 and Tie2.

In this report we demonstrate that Thr794 of Tie1 is critical for angiogenesis and vascular morphogenesis *in vivo* during zebrafish vascular development and *in vitro* in matrigel tube formation assays. We demonstrate that these effects are mediated in part by a novel interaction between Tie1 and the small GTPase Rac1 that requires Rac1-PAK1-mediated phosphorylation of Tie1-T794. These findings demonstrate a novel signaling pathway involving Tie1 that is required for angiogenesis.

## Methods

### Cell lines and Reagents

Rabbit polyclonal phospho-Tie1-T794 antibody was generated by 21^st^ Century Biochemicals (Marlborough, MA) using the peptide sequences C-Ahx-LHRRRTF[pT]YQSGSGE-amide & C-Ahx –LHRRR[pT]F[pT]YQSGSGE-amide. Rabbit polyclonal Tie1 C-tail antibody (C–18, SC–342), mouse monoclonal phospho-Tyrosine antibody (PY99, SC–7020), and rabbit polyclonal Tie2 C-tail antibody (C–20, SC–324) were from Santa Cruz Biotech (Santa Cruz, CA). Extracellular Tie2 mouse monoclonal antibody (mAb 33) was from Amgen (Thousand Oaks, CA). Rabbit polyclonal phospho-Akt substrate (#10001), phospho-Akt (Ser473) (#9271), total Akt (#9272), total p44/42 MAPK (ERK1/2, #9102), Rac 1,2,3 (#2465), phospho-PAK1 (S144)/ PAK2 (S141) (#2606), PAK1 (#2602), and mouse P-p44/42 (#9106) were from Cell Signaling Technology (Danvers, MA). Rat alpha-Tubulin (#MCA77G) antibody was from AbD Serotec (Raleigh, NC). Rho/Rac/Cdc42 activator (#CN04), recombinant His-Rac1 protein (#RC01) and ROCK inhibitor Y–27632 (#CN06) were from Cytoskeleton Inc (Denver, CO). PAK inhibitor IPA 3 (#3622) was from Tocris Biosciences (Minneapolis, MN). Complete^®^ Protease inhibitor and PhosStop^®^ phosphatase inhibitor were from Roche (San Francisco, CA). HUVECs were isolated in lab from umbilical cords donated to the Duke University Cord Blood Bank as previously described [16]. Human embryonic kindney (HEK)-293 cells were purchased from ATCC (Manassas, VA). Adenoviruses were generated using the pAdTrack-CMV/pAdEasy system as previously described [17]. Matrigel was from BD Biosciences/Corning (#354234) (Corning, NY). HUVECs were grown on 0.1% gelatin-coated dishes in Lonza (Basel, Switzerland) EBM (#CC–3121) supplemented with a Lonza EGM Singlequot (#CC–4133) with 1% pen/strep and 20% FBS.

### *In vitro* kinase assays

0.5–1.0μg of GST-Tie kinase fusion protein was precipitated from SF9 insect cell lysates using 30ul Glutatione-sepharose beads (Amersham Biosciences, Pittsburgh, PA). Purified proteins were washed in Triton lysis buffer (20mM Tris (pH 8.0), 137mM NaCl, 2mM EDTA (pH 8.0), 10% v/v Glycerol, 1% v/v Triton-X, Complete^®^ protease inhibitor tablets, PhosSTOP^®^ phosphatase inhibitor) and incubated with 1mM ATP, kinase buffer (20mM Tris-HCL, pH 7.5, 100uM NaCl, 12mM MgCl_2_, 1mM DTT) and endothelial cell lysate for 1hr at RT with mixing. The GST-beads were then washed twice more with lysis buffer before the proteins were eluted into Laemmli buffer to be resolved by SDS-PAGE and western blot.

### Morpholino and embryo manipulations

All zebrafish embryo studies were approved by the Duke University Institutional Animal Care and Use Committee. Zebrafish (*Danio rerio*) embryos were raised and maintained as described [18]. 24 hours post-fertilization (hpf) embryos were raised in 0.2 mM 1-phenyl-2-thio-urea (Sigma) to prevent pigment formation and were allowed to develop until 48 hpf. Splice blocker morpholino (MO) against *tie1* (5’-CATGTCTACTTACAGATCCAGATTG–3’) was reported previously [19] and was obtained from Gene Tools, LLC.

1nl of diluted MO (5 ng) and/or RNA (50 or 100 pg) was injected into Fli1:EGFP zebrafish embryos at the 1- to 2-cell stage. Injected embryos in each group were blinded by a member of the Katsanis lab not involved with the project and classified at 48 hpf (by C. Golzio) as normal or mutant based on relative vasculogenic defects compared with age-matched controls from the same clutch. Mutant embryos were characterized by a minimum of four missing intersegmental vessels (ISV) and/or the absence of the dorsal longitudinal anastomotic vessel (DLAV). For RNA rescue experiments, the murine Tie1-wild type and Tie1*-*T794A mRNAs were cloned into the pCS2 vector and transcribed *in vitro* using the SP6 Message Machine kit (Ambion). Numbers of embryos per group were as follows: Uninjected, n = 300; Control MO, n = 62; Tie1-MO (MO), n = 133; MO+Tie1-WT, n = 105; MO+ Tie1-T794A, n = 79; Tie1-T794+WT, n = 27; Tie1-WT, n = 275; Tie1-T794A, n = 60.

### Western blot analysis

HUVECs were infected with GFP or Tie1 adenoviruses at ~3x10^8^ pfu/ml +/- AdmyrAkt and incubated overnight. Ligand stimulation experiments were starved for 3h prior to incubation with ligand as indicated. All CN04-mediated Rac1 activation experiments were starved overnight followed by a 30 min pre-treatment with inhibitors and/or 3h CN04 (5μg/ml) incubation. Cells were then washed once with PBS and lysed in Triton lysis buffer containing protease and phosphatase inhibitors. Lysates and immunoprecipitates were resolved by SDS-PAGE and analyzed by western blot.

### Matrigel tube formation assay

HUVECs were infected overnight with GFP or Tie1 adenoviruses at ~3x10^8^ pfu/ml. Cells were then plated on Matrigel (10mg/ml) at 6,000 cells/well of a 96-well plate. Remaining cells were lysed and analyzed by western blot to assure equal adenoviral Tie1 expression. Brightfield images were captured using an inverted epi-fluorescent (6x objective) microscope 6 hours after plating and quantified using ImageJ-Angiogenesis Analyzer macro. Fluorescent images were taken by fixing tubes in 4% PFA, permeabilizing in 0.5% Triton-X, and staining with 70nM Rhodamine-Phalloidin.

### Tie1 Proteomic association

Tie1 antibody (C–18, Santa Cruz) was covalently conjugated to Protein-A agarose beads using a Dimethylpimelimidate (DMP)-crosslinking protocol provided by the Duke Proteomics Core Facility. In brief, antibody was coupled to agarose beads by mixing for several hours. Beads were then washed 3x in a 0.2M sodium borate buffer (pH 9.0) to remove uncoupled antibody. Antibody was cross-linked using freshly made 20mM DMP in 0.2M sodium borate (pH 9.0) for 40 minutes at room temperature. Beads were then washed in 0.2M ethanolamine to quench the DMP reaction, then subjected to an acid wash (0.58% v/v acetic acid + 150 mM NaCl) 3x to remove uncoupled antibody. Coupled beads were then validated for efficiency and specificity by Coomasie stain and Western blot, respectively.

Immortalized endothelial cells (ECRFs) constitutively expressing a human Tie1 shRNA were infected with adenoviruses expressing mouse Tie1-WT or T794A +/- AdmyrAkt for 24 hours then lysed in NP–40 lysis buffer. Tie1 was immunoprecipitated on cross-linked beads and washed thoroughly with NP–40 lysis buffer. Samples were then washed 3x in 50mM Ammonium Bicarbonate and mixed with 0.2% Rapigest SF Surfactant (Waters Corporation) and delivered to the Duke Proteomics Core Facility for in solution digestion and proteomic analysis of associated proteins. Unique peptides were identified using the SwissProt database. Peptides that bound to the IgG-beads control or known to non-specifically bind to the Protein A/G agarose beads were discarded as non-specific. Proteins identified by 2 or more unique peptides were considered to be specific binders.

### Co-Immunoprecipitation

HUVECs were infected with GFP or Tie1 adenoviruses and lysed in NP–40 lysis buffer (100mM NaCl, 1.0% NP–40, 50mM Tris-Cl, pH8.0,) containing protease and phosphatase inhibitors. Lysates were incubated on a rotator at 4°C with 1ug of recombinant His-Rac1 for 6 hours. Aliquots of whole cell lysate were collected at this point to analyze by Western blot. The remaining lysate was mixed with Tie1 antibody overnight. Protein-A Agarose beads were added for 1 hour the following morning. Proteins precipitated on the beads were then washed 3 times with NP–40 lysis buffer and mixed with 1× Laemmli running buffer containing 5% β-mercaptoethanol, boiled for 5 minutes, and resolved by SDS-PAGE.

### Proliferation assay

HUVECs were infected with the indicated viruses overnight then plate at 10,000 cells/well of a 6-well plate. Every 24 hours following plating, separate groups of cells were fixed in cold methanol for 5 minutes, then allowed to dry before staining with 50% hematoxylin. Four random images were captured per well and number of nuclei were counted. Cell numbers were normalized to the mean number of cells 24 hours after plating.

### Survival Assay

Nearly-confluent HUVECs were left uninfected or were infected with the indicated adenovirus. 24 hours after infection, the media was changed to either full-serum media or serum- and growth factor-free starvation media. 48 hours later, the cells were washed once with PBS to remove dead and non-adherent cells, fixed in methanol, hematoxylin stained, and random fields were imaged with a 6× objective. Nuclei were counted and normalized to the average number of cells in the full-serum media group for each virus condition to account for any proliferation that may have taken place.

### Leukocyte Adhesion Assay

Confluent HUVECs were infected for 24 hours with the indicated adenovirus, then treated with or without TNFα (1ng/ml) overnight. U937 leukocytes (3x10^6^) were then incubated with the HUVECs for 30 minutes at 37°C. Non-adhered cells were washed away. Brightfield images (4x objective) were captured and adherent U937s were counted.

### Migration Assay

HUVECs were infected with the indicated adenovirus for 24 hours then plated at 30,000 cells/well in serum-free media on to 8μm Transwell filters (Corning) coated with gelatin (0.1%). Full-serum growth media was added to the lower chamber and cells were allowed to migrate for 6 hours. Unmigrated cells were then removed from the top of the membrane using a cotton swab. Migrated cells were fixed in methanol for 15 minutes and incubated with a DAPI solution (300nM in PBS) for 5 minutes. Random fields were chosen and counted.

### Statistical Analysis

All western blots are representative of at least 3 repeat experiments. Quantification of tube formation data was the result of 11 independent experiments, each normalized to uninfected controls for comparison. Zebrafish data are expressed as a percentage of the total number of embryos counted per group. Significance was determined by a Chi squared test. All other results are expressed as the mean ± the standard error of the mean (SEM). Analysis of variance was used to compare means of different groups and Student’s t-tests were used to determine individual differences. In all instances, P<0.05 was considered statistically significant.

## Results

### Tie1 can be phosphorylated by Akt on Thr794

**Fig 1:**
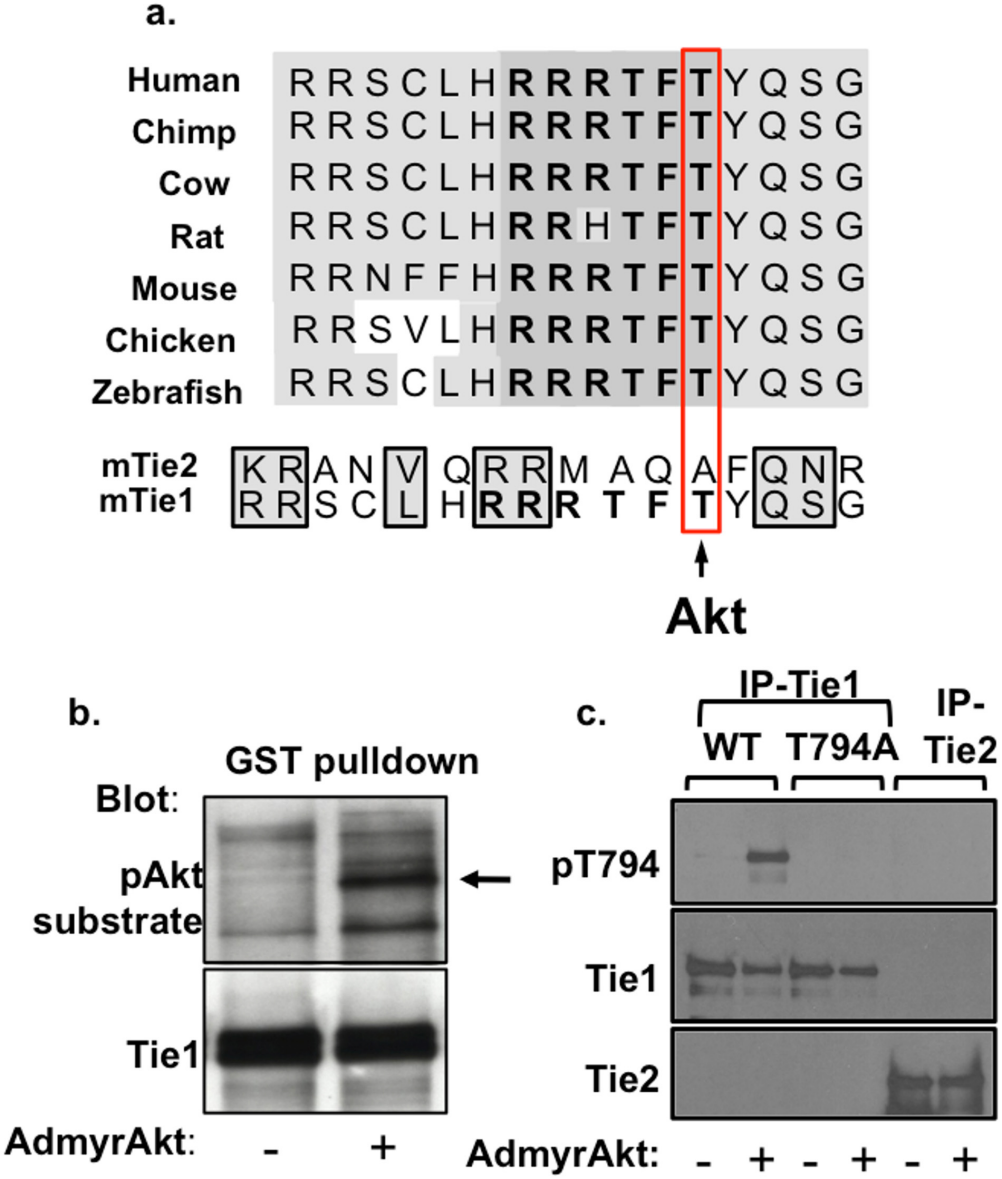
Tie1 contains an Akt consensus phosphorylation site and can be phosphorylated *in vitro* by Akt. **a** The juxtamembrane (JM) domain of Tie1 is highly conserved from zebrafish through humans (light gray), including a high probability Akt phosphorylation consensus sequence (RRRTFTY) within the JM region (dark gray). The predicted phosphorylation site at Tie1-Thr794 is not present in Tie2. **b** GST-Tie1 fusion protein was incubated with EC lysates that had been infected with AdEmpty (-) or AdmyrAkt (+) virus in an *in vitro* kinase reaction. Phosphorylated Tie1 (arrow) was detected with a phospho-Akt substrate antibody. **c** HUVECs expressing Tie1WT, -T794A, or Tie2 were infected with AdEmpty (-) or AdmyrAkt (+) and Tie1 or Tie2 was immunoprecipitated (IP) and probed with a phospho-specific Tie1-pT794 antibody.

The presence of a large number of Ser/Thr residues in the Tie1 JM region suggested that it might be phosphorylated on one or more of these residues as a means of regulating its activity and/or function. A Scansite motif search revealed that Thr794 (mouse protein sequence) lies within a high-probability Akt consensus phosphorylation sequence (Fig 1a). To test whether Akt could phosphorylate Tie1, we performed *in vitro* kinase reactions using a fusion protein comprised of the Tie1 JM and kinase domains fused to GST (GST-Tie1) incubated with endothelial cell lysates that had been infected with either a control, empty adenovirus (AdEmpty) or constitutively active, myristoylated Akt adenovirus (AdmyrAkt). Western blotting with a phospho-Akt-substrate antibody, which detects the RXRXXpS/T sequence on many Akt substrates, demonstrated that Tie1 was phosphorylated after incubation with lysates from AdmyrAkt infected cells but not AdEmpty infected cells (Fig 1b). To validate these findings, we then developed a rabbit polyclonal phospho-T794 antibody as well as adenoviruses encoding wild-type (WT) Tie1 and a non-phosphorylatable T794A mutant. Human umbilical vein endothelial cells (HUVECs) were infected with AdEmpty or AdmyrAkt, and Tie1 or Tie2 was immunoprecipitated (IP’ed) and probed with the Tie1-pT794 antibody. Consistent with our earlier result, Tie1-WT, but not Tie2, was phosphorylated in the presence of active Akt, and this effect was blocked by mutating Tie1-Thr794 to alanine (Fig 1c).

### Tie1-T794 plays a critical role in zebrafish vascular development

**Fig 2:**
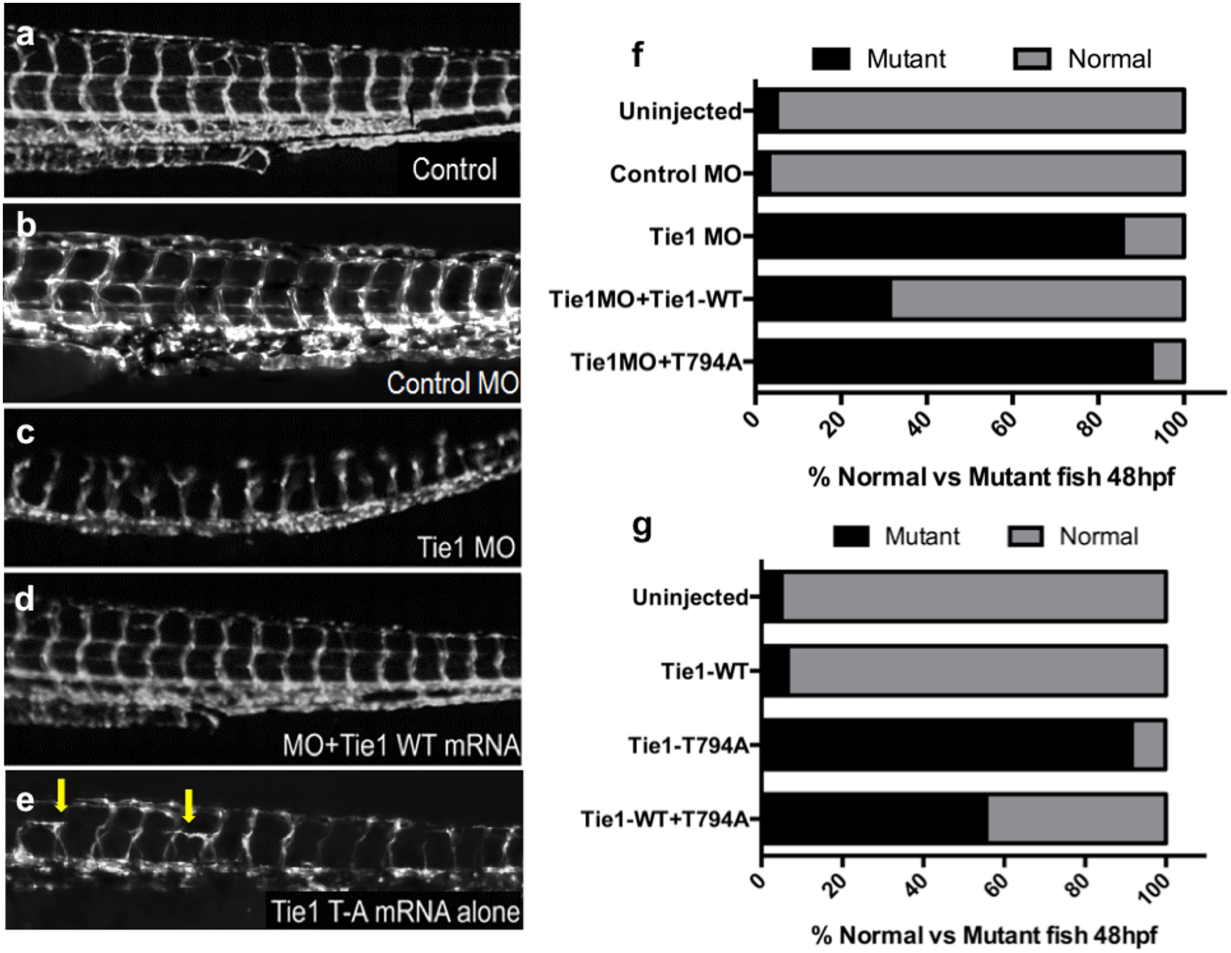
Tie1 and Thr794 are required for proper embryonic vascular development in zebrafish. **a-e** Zebrafish embryos expressing Fli1:EGFP were injected with a control or Tie1 morpholino (MO) with or without mRNA encoding WT or T794A (TA) mutant Tie1 and effects on vascular development were assessed. **b** Zebrafish injected with a control MO develop normally and resemble uninjected (Control) embryos. **c** Tie1 MO-injected embryos display vascular abnormalities. **d** Co-injection of Tie1 MO and WT mouse Tie1 mRNA rescued the MO phenotype. **e** Injection of T794A mutant mouse Tie1 mRNA alone resulted in vascular abnormalities similar to those of MO-injected embryos. Arrows indicate blunted intersomitic vessel formation. **f** Quantification of mutant embryos after injection of Tie1 MO with WT or T794A mutant mRNA, P = 3.0x10^-19^ by Chi squared analysis. **g** Quantification of mutant embryos after injection of Tie1-WT or -TA mRNA alone, P = 1.3x10^-18^ by Chi squared analysis.

To assess the biological significance of Tie1 Thr794 as a potential phosphorylation site during vascular development, we utilized a Fli1:EGFP zebrafish transgenic line, which expresses GFP in endothelial cells (ECs) (Fig 2a). A validated splice-blocking morpholino (MO) [19] for the sole ortholog of Tie1 in zebrafish was used to perform knockdown experiments. Zebrafish embryos at the one- to two-cell stage were injected with 5ng of Tie1 or control MO and raised for 48 hours post-fertilization. Abnormal vascular development was then characterized by aberrant formation of intersomitic and dorsal longitudinal anastomotic vessels. As expected, morpholino (MO)-mediated knockdown of Tie1 resulted in abnormal vascular development (114/133 mutant; Fig 2c). Uninjected embryos as well as embryos injected with a control MO were also assessed as baseline controls and showed minimal vascular abnormalities (15/300 mutant, 2/62 mutant, respectively). To test whether this phenotype is specific to Tie1 and not due to off-target effects of the MO, *in vitro* transcribed capped mRNA encoding the WT mouse Tie1 was co-injected together with Tie1 MO, which significantly rescued the morphant phenotype (33/105 mutant, p<0.001; Fig 2d). Subsequently, *in vivo* complementation with mRNA encoding the non-phosphorylatable T794A mutant was performed. The T794A mutant failed to rescue MO-mediated aberrant vascular development, and the embryos were indistinguishable from morphants (73/79 mutant; Fig 2e and 2f). Moreover, abnormal vascular development similar to that in MO-injected embryos was observed in those injected with the Tie1-T794A mutant alone (55/60 mutant), whereas injection of Tie1-WT alone did not disrupt vascular development (18/293 mutant; Fig 2g). Because the T794A mutant could be inappropriately dampening or augmenting the angiogenic signaling pathways, both possibilities could manifest as the observed “mutant” phenotype in these assays. Therefore, to test whether the T794A mutant acts as either a dominant negative or a gain-of-function allele, T794A mutant mRNA was titrated with Tie1-WT mRNA, which significantly ameliorated the aberrant phenotype (15/27 mutant; Fig 2f), supporting the notion that the T794A mutant acts as a dominant negative inhibitor of developmental angiogenesis. Taken together, our *in vivo* data suggest that the integrity of Tie1-Thr794 is required for proper sprouting of intersomitic vessels and that an unphosphorylatable Tie1 mutant can act as a dominant negative inhibitor during angiogenesis (Fig 2f).

### Tie1-T794 is required for endothelial tube formation

**Fig 3:**
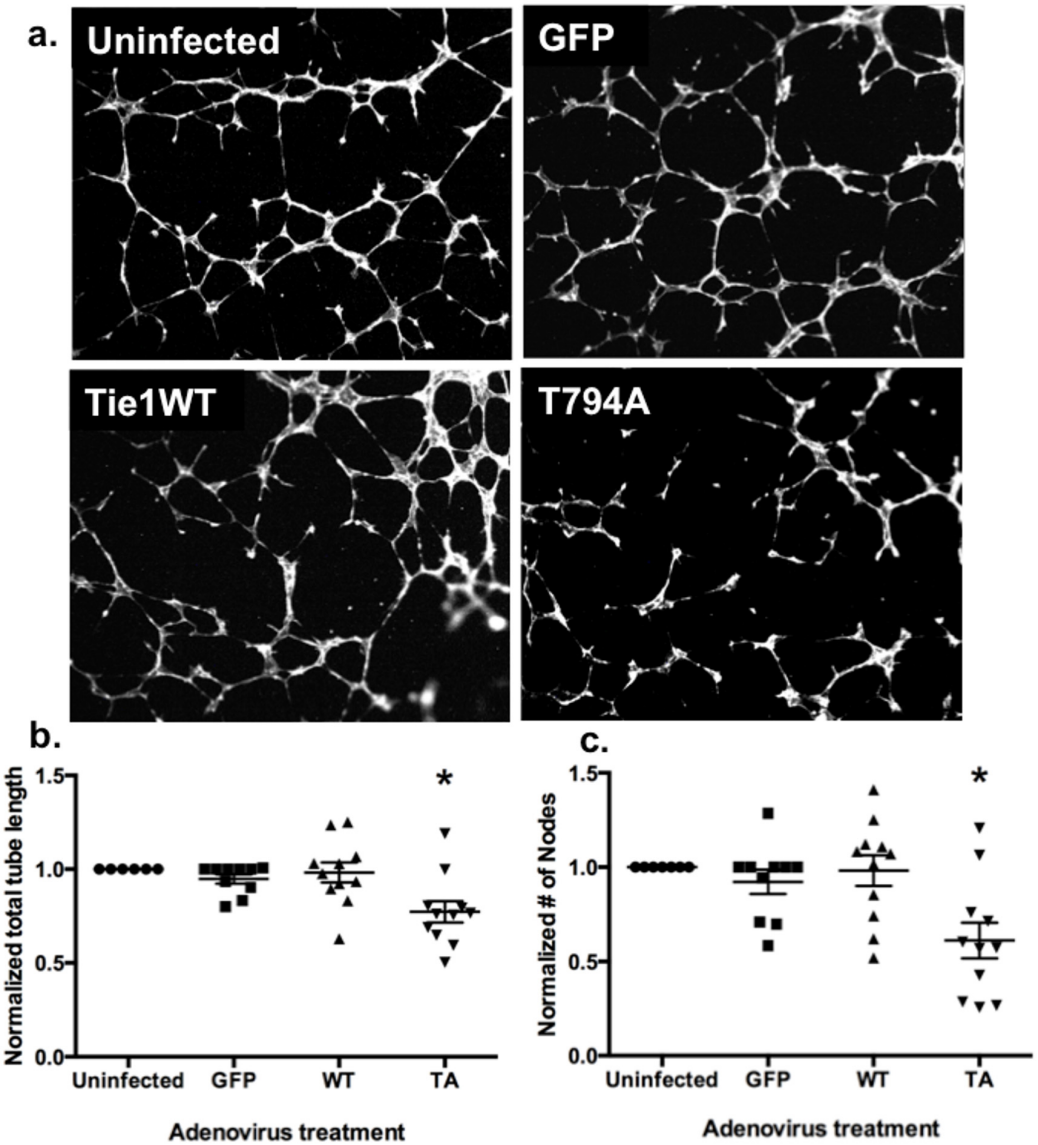
The Tie1 T794 mutant disrupts endothelial tube formation. **a** Representative images of HUVECs left uninfected or infected with adenoviruses encoding GFP, Tie1-WT, or Tie1-T794A, plated on Matrigel and allowed to form tubes for 6 hours. **b, c** Quantification of endothelial tube networks *in vitro*. Cells expressing the T794A mutant formed significantly shorter tubes (**b**; *, multiple comparisons P≤ 0.02) with fewer nodes and branches (**c**; *, multiple comparisons P< 0.01) (n = 11 independent experiments with 4 or more images per experiment).

Adenoviruses encoding Tie1-WT or -T794A were used to study the potential role of T794 phosphorylation *in vitro*. HUVECs infected with these adenoviruses were plated on Matrigel and allowed to form tubes for 6 hours before fixation, phalloidin staining, and imaging (Fig 3a). Cells infected with the T794A mutant formed tubes that were significantly shorter and significantly less branched than those that were left uninfected or expressing GFP or Tie1-WT (Fig 3b and 3c) (P<0.05, multiple comparisons). Interestingly, expression of the T794A mutant also significantly decreased EC migration compared to unifected HUVECs (S1 Fig) (P<0.001).

### The T794A mutation does not alter Tie1 tyrosine phosphorylation or Ang1-mediated Tie2 signaling

Based on the effects of the T794A mutant on vascular development *in vivo* and on tube formation *in vitro*, we speculated that this site might be required for signaling by Tie1 and/or Tie2. We and others have shown that the Tie1 kinase can transduce signals, albeit weakly [20, 21], although investigating effects on Tie1 activity is complicated by its inability to directly bind ligands. However, Tie1 can be activated by Ang1 in the presence of Tie2 [8]. When HUVECs expressing Tie1-WT or -T794A were treated with Ang1, no appreciable differences in tyrosine phosphorylation of Tie1 or Tie2 were observed, and Ang1-mediated activation of Akt and ERK remained unchanged (S2 and S3 Figs). These results suggested that the effects of the T794A mutant on angiogenesis are not a result of changes in Tie1 or Tie2 kinase activation. Additionally, Tie1’s known pro-inflammatory role in ECs was unaffected by expression of the T794A mutant when assessed by leukocyte adhesion (S4 Fig). Physiological functions commonly associated with Tie2 activation such as survival (S5a Fig) and proliferation (S5b Fig) were also unaffected by T794A expression. Interestingly, overexpression of Tie1-WT did decrease EC proliferation, but in a T794-independent manner (S5b Fig). Physiological activation of Akt by Ang1, Ang2, or VEGF resulted in no detectable phosphorylation of Tie1 on T794 (S6 Fig), suggesting that Akt may not phosphorylate Tie1-T794 under physiological circumstances.

### PAK1-mediated Tie1-T794 phosphorylation is required for interaction with Rac1

**Fig 4:**
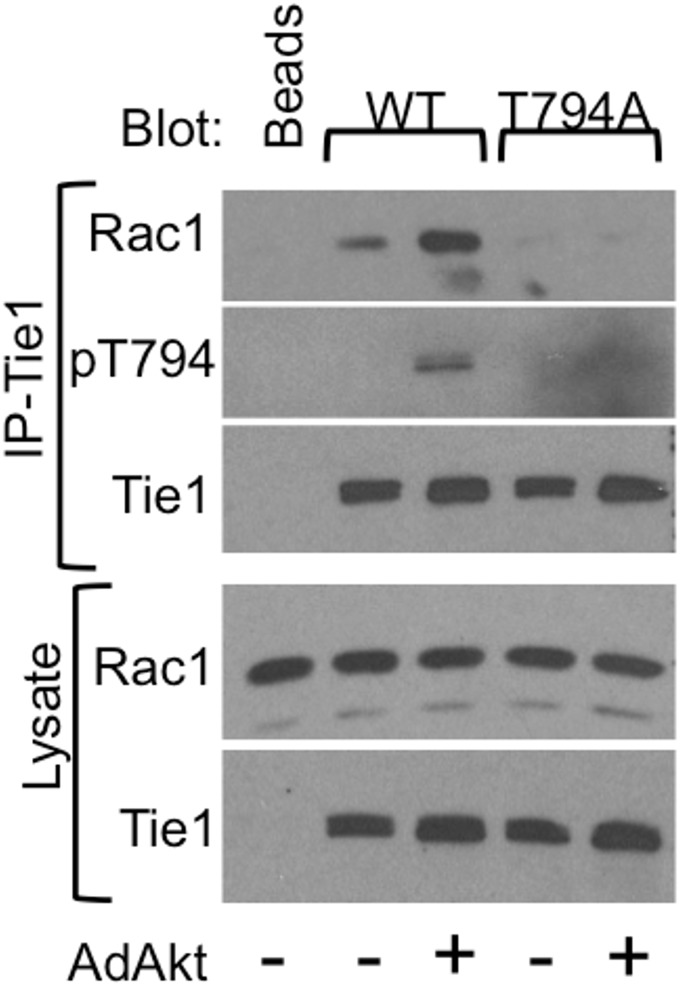
Rac1 association with Tie1 is enhanced by T794 phosphorylation. HUVECs were infected with adenoviruses encoding Tie1-WT and -T794A, with or without AdAkt, and Tie1 was immunoprecipitated (IP) from cell lysates and western blotted as indicated. Whole cell lysates were blotted for total Tie1 and Rac1.

To further investigate mechanisms by which mutation of T794 contributes to defects in angiogenesis, we performed a mass spectrometry-based analysis of Tie1-associated proteins using Tie1-WT or Tie1-T794A in the presence or absence of AdmyrAkt to distinguish between T794-dependent and -independent Tie1 protein interactions (raw data in Supporting Information). Mass spectrometry revealed a number of Tie1-interacting proteins (S1 Table). Among these we focused on the small GTPase Rac1 for two reasons: its apparent dependence on T794 phosphorylation and its known role in regulation of the cytoskeleton, angiogenesis, and vascular morphogenesis [22–24]. To validate the association of Rac1 with Tie1, HUVECs were infected with adenoviruses encoding Tie1-WT or -T794A with or without AdmyrAkt to induce T794 phosphorylation, and Tie1 was IP’ed and resolved by SDS-PAGE and blotted for Rac1 (Fig 4). Rac1 strongly associated with Tie1-WT in the presence of phospho-T794, but no associated Rac1 was detected with the T794A mutant, suggesting that phospho-T794 may serve as a docking site for Rac1.

**Fig 5:**
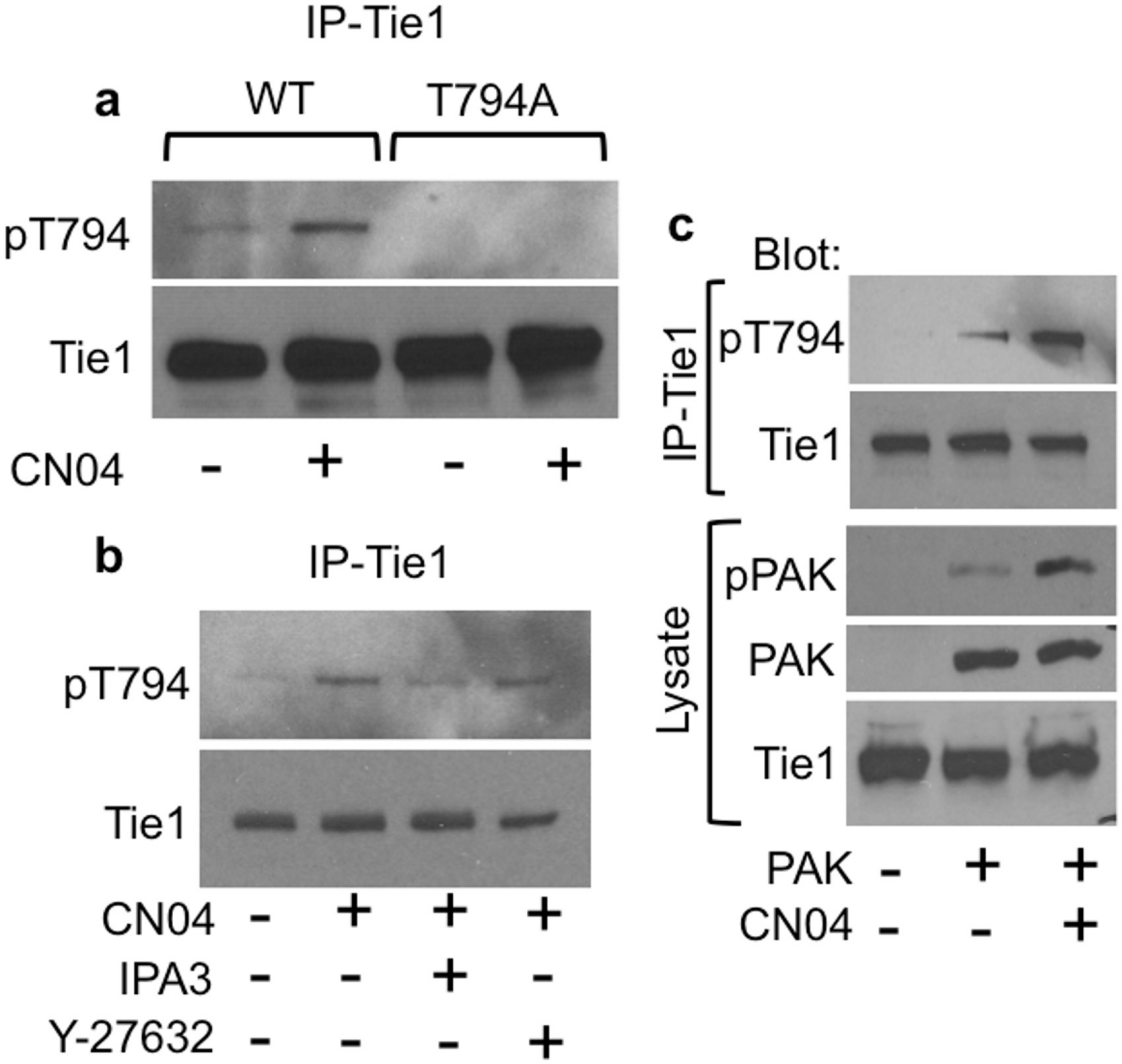
Rac1-mediated PAK1 activation results in Tie1-T794 phosphorylation. **a** Rac1 was activated using CN04 in HUVECs infected with AdTie1 WT or T794A. Tie1 was immunoprecipitated (IP) and western blotted as indicated. CN04-mediated Rac1 activation resulted in T794 phosphorylation which was ablated by the T794A mutant. **b** CN04-mediated T794 phosphorylation was reduced by PAK1 inhibition (IPA3, 10μM) but not ROCK1 inhibition (Y–27632, 10μM). **c** HEK-293T cells transfected with Tie1-WT alone or together with PAK1 were treated with or without CN04, and Tie1 IPs and whole cell lysates were western blotted as indicated. Co-expression of PAK1 induced Tie1-T794 phosphorylation, which was augmented by CN04-mediated activation of Rac1 and PAK1.

Given our negative results with any physiological activators of Akt to induce T794 phosphorylation, the interaction of Tie1 with Rac1 raised the possibility of a feedback loop involving effector kinases downstream of the Rho GTPases, such as PAK1 and ROCK. Therefore, we assessed these serine/threonine kinases for their potential ability to phosphorylate Tie1-T794. Treatment of HUVECs with CN04, a Rho/Rac/Cdc42 activator that induces activation of both PAK1 and ROCK, resulted in readily detectable T794 phosphorylation, which was abrogated by expression of the T794A mutant (Fig 5a). To determine which downstream kinase is responsible for T794 phosphorylation, HUVECs were pretreated with IPA3, a PAK inhibitor, or Y–27632, a ROCK inhibitor, for 30 minutes prior to CN04-mediated Rac1 activation. We observed a reduction in T794 phosphorylation in the presence of IPA3 but little change after ROCK inhibition, suggesting that PAK1 is responsible for T794 phosphorylation (Fig 5b). To confirm this finding, we transfected Tie1 into HEK–293 cells alone or together with PAK1. Whereas phospho-T794 was undetectable at baseline, co-expression of PAK1 resulted in readily detectable phospho-T794 and phospho-PAK1. Moreover, activation of Rac1 by the addition of CN04 resulted in an increase in phosphorylation of both Tie1 and PAK1 (Fig 5c).

## Discussion

An essential role for Tie1 in embryonic vascular development was demonstrated by two separate groups 20 years ago. Recent studies in conditional Tie1 knockout mice *in vivo* and Tie1 knockdown or overexpression in ECs *in vitro* have provided some functional insights, however these studies have provided little clarity regarding Tie1’s mechanism of action. For instance, Tie1 overexpression *in vitro* results in upregulation of inflammatory genes such as ICAM, VCAM, and VE-Cadherin, whereas silencing Tie1 in ECs decreases expression of these inflammatory genes and proteins. Accordingly, EC-specific conditional deletion of Tie1 limited vascular inflammation and the development of atherosclerosis in ApoE^-/-^ mice [25]. More recently, conditional EC-specific Tie1 deletion was shown to inhibit tumor growth and angiogenesis as well as retinal neovascularization [26]. Decreased vascularity in the eye after loss of Tie1 was linked to Notch activation, and inhibition of Notch signaling rescued the angiogenic defect caused by loss of Tie1. However, the molecular mechanisms by which Tie1 mediates its effects on inflammation and angiogenesis remain completely unknown. In this report, we provide the first demonstration of a signaling pathway mediated by Tie1 that is required for angiogenesis, which is an important first step in elucidating Tie1’s role in vascular growth and development.

Tie1 is generally still classified as an orphan receptor, as it is unable to bind directly to any known ligands, including the angiopoietins, the ligands for Tie2. Saharinen et al demonstrated that Tie1 could be activated by Ang1, and receptor activation (tyrosine phosphorylation) was enhanced in the presence of Tie2, suggesting that Tie1 could serve as a co-receptor for Tie2 and possibly other receptors, such as integrins, which play a role in angiopoietin-Tie2 signaling [27–29]. Crystallography data have provided a structural basis for Tie1’s interaction with Tie2, which is mediated by electrostatic interactions between the two receptors’ extracellular domains in the absence of ligand [7, 30]. Although evidence demonstrates that Tie1 negatively regulates Tie2 signaling [8] and this may be facilitated by protease-mediated cleavage and shedding of the Tie1 extracellular domain [31], signaling pathways downstream of Tie1 have been largely unstudied, in part because of the inability to investigate ligand-mediated receptor activation and signaling.

To circumvent this problem, our group generated a CSF–1 receptor-Tie1 chimera and demonstrated that the Tie1 kinase could be ligand-activated and transduce cell survival signals through PI 3-kinase and Akt [20]. Consistent with these findings, conditional deletion of Tie1 resulted in increased EC apoptosis in mice [26]. Our current studies provide evidence for a novel pathway downstream of Tie1 that is necessary for both sprouting angiogenesis and capillary morphogenesis. However, the mechanisms responsible for activation of this Rac1/PAK1/Tie1 pathway remain unclear. Although we cannot rule out a role for angiopoietin-mediated Tie1 phosphorylation in our zebrafish and matrigel studies, this seems unlikely given that Ang-1-treatment of ECs failed to induce detectable Tie1-T794 phosphorylation. Moreover, the fact that our most robust findings were observed *in vivo* in zebrafish and in matrigel suggests an important role for cell-matrix interactions, such as those mediated by integrins, although this remains to be proven. Taken together, however, our results suggest that Tie1 may not act as a canonical RTK, but perhaps more like the EGF receptor ErbB2, which does not bind ligand but is able to multimerize with and modulate the signaling of the other EGF receptors [32]. In addition to its modulatory effects on Tie2, Tie1 might act as a molecular scaffold, particularly after phosphorylation of T794 and/or other Ser/Thr residues in the JM region, facilitating interaction with Rac1 and other proteins, including other small GTPases, to regulate downstream endothelial cell functions.

Using the zebrafish model, we were able to assess the biological importance of the putative Akt consensus phosphorylation site at Thr794. In support of the specificity of our approach, knockdown of Tie1 resulted in defects in angiogenesis and vascular integrity similar to those shown previously in mice [4, 5, 33, 34] as well as those observed in a previous characterization of the Tie1 MO [19], and we were able to rescue this phenotype by co-expression of mammalian (murine) wild-type Tie1. Strikingly, not only was the T794A point mutant unable to rescue the angiogenesis defects, but it also induced an apparent dominant negative inhibitory effect on angiogenesis during zebrafish vascular development. Since we were unable to detect *in vivo* T794 phosphorylation, we cannot rule out the possibility that the T794A mutant affects vascular development in a phosphorylation-independent manner. It is unlikely that the T794A point mutation would adversely affect translation or proper folding of the Tie1 protein, as the same point mutant was readily expressed *in vitro* and behaved functionally similar to Tie1-WT in non-angiogenic contexts. However, it is possible that it could alter protein-protein interactions in a phosphorylation-independent manner. Nonetheless, our *in vivo* findings, together with complementary *in vitro* tube formation assays, Rac1/PAK1-mediated T794 phosphorylation studies, and Rac1’s known role in sprouting angiogenesis strongly suggest that the ability to modulate the phosphorylation state of T794 may be critical for proper angiogenesis and capillary morphogenesis. These data also suggest a role for Tie1 in modulation of the endothelial cytoskeleton, a role that has not been demonstrated previously.

Ser/Thr phosphorylation has been demonstrated for a number of RTKs and has been shown most commonly to negatively regulate kinase activity and downstream signaling [12–14]. Somewhat surprisingly, however, we were unable to demonstrate an effect of the T794A mutant on any of the known functions of Tie1, including Tie1 tyrosine phosphorylation, inflammatory leukocyte adhesion, or Ang1-mediated Tie2 phosphorylation, signaling, and cell survival. This raised the possibility that phospho-T794 might serve as a docking site for protein interactors, which led us to perform a proteomic analysis of Tie1-associated proteins. Of the 26 specific interacting proteins identified by this screen, four were found to be small GTPases of the Ras superfamily, which play well documented roles in endothelial cell sprouting and tube formation [22, 35], and which would be consistent with a role for Tie1-pT794 in cytoskeletal regulation. Notable among these was Rac1, a member of the Rho-family of GTPases, as it appeared to bind to Tie1 only in the presence of Akt, and thus presumably when T794 was phosphorylated. This possibility was validated by the fact that Rac1 co-immunoprecipitated with Tie1-WT but not with the Tie1-T794A mutant (Fig 4).

Like most small GTPases, Rac1 cycles between an active GTP-bound state and an inactive GDP-bound state. Basal Rac1 signaling generally mediates endothelial cell barrier protection, whereas Rac1 activation promotes intercellular attachments and regulation of actin and microtubule rearrangement during endothelial lumen formation [36]. As with many factors that are critical for angiogenesis, increases or decreases in Rac1 activity can result in vascular abnormalities [1–3, 33]. Rac1 knockout has been shown to inhibit EC migration and tubulogenesis [23], and mice with endothelial Rac1 haploinsufficiency display mild hypertension that appears to be a result of insufficient endothelial nitric oxide synthase (eNOS) activity [37]. Conversely, sustained Rac1 signaling has been shown to generate an abundance of reactive oxygen species, resulting in vascular smooth muscle hyperplasia [38]. In this regard, it remains unclear from our studies whether T794 is required for Rac1 activation or to limit Rac1 signaling. In either case, it is clear that T794 is required for proper angiogenesis and tube formation.

Considering that we suspected T794 to be an Akt phosphorylation site, the lack of an effect of the T794A mutant on cell survival and proliferation was particularly surprising and raised the question as to whether another kinase might be involved. Although the RRRTFTY sequence in the Tie1 JM domain could be phosphorylated by overexpression of constitutively active Akt, we were unable to identify a physiological stimulus that induced Akt-mediated phosphorylation of T794, raising concerns about promiscuity following overexpression of constitutively active Akt [39]. While our results do not rule out the possibility that Akt could phosphorylate Tie1 *in vivo*, neither can we confirm Tie1 as an Akt substrate as defined by Manning and Cantley [39]. While investigating the interaction between Tie1 and Rac1, we discovered that we could induce T794 phosphorylation with CN04, a cell-permeable deaminase that blocks intrinsic and GTPase-activating protein (GAP)-mediated GTP hydrolysis, effectively resulting in constitutive activation of Rac1, RhoA, and Cdc42. This led us to examine the serine/threonine kinases activated downstream of these small GTPases as potential Tie1-T794 kinases, namely Rho-kinase (ROCK1) and p21-activated kinase–1 (PAK1). ROCK1 recognizes the consensus sequences R/KXS/T and R/KXXS/T and therefore could potentially phosphorylate Tie1 on T792 (RRT) or T794 (RTFT) [40]. According to a study by Rennefahrt et al., PAK1 predominately favors arginine residues in the -5 to -1 positions, and the triplet arginines in the -5 to -3 positions upstream of T794 would make this a favorable PAK1 phosphorylation site. Additionally, bulky hydrophobic residues (Val, Ile, Trp, and Tyr) are favored in the +1 to +3 positions, thus the tyrosine residue in the +1 position would further favor PAK1-mediated phosphorylation of T794 [41]. Our results with pharmacological inhibitors of these two kinases strongly indicated that PAK1 is responsible for phosphorylation of Tie1-T794 downstream of Rac1.

**Fig 6:**
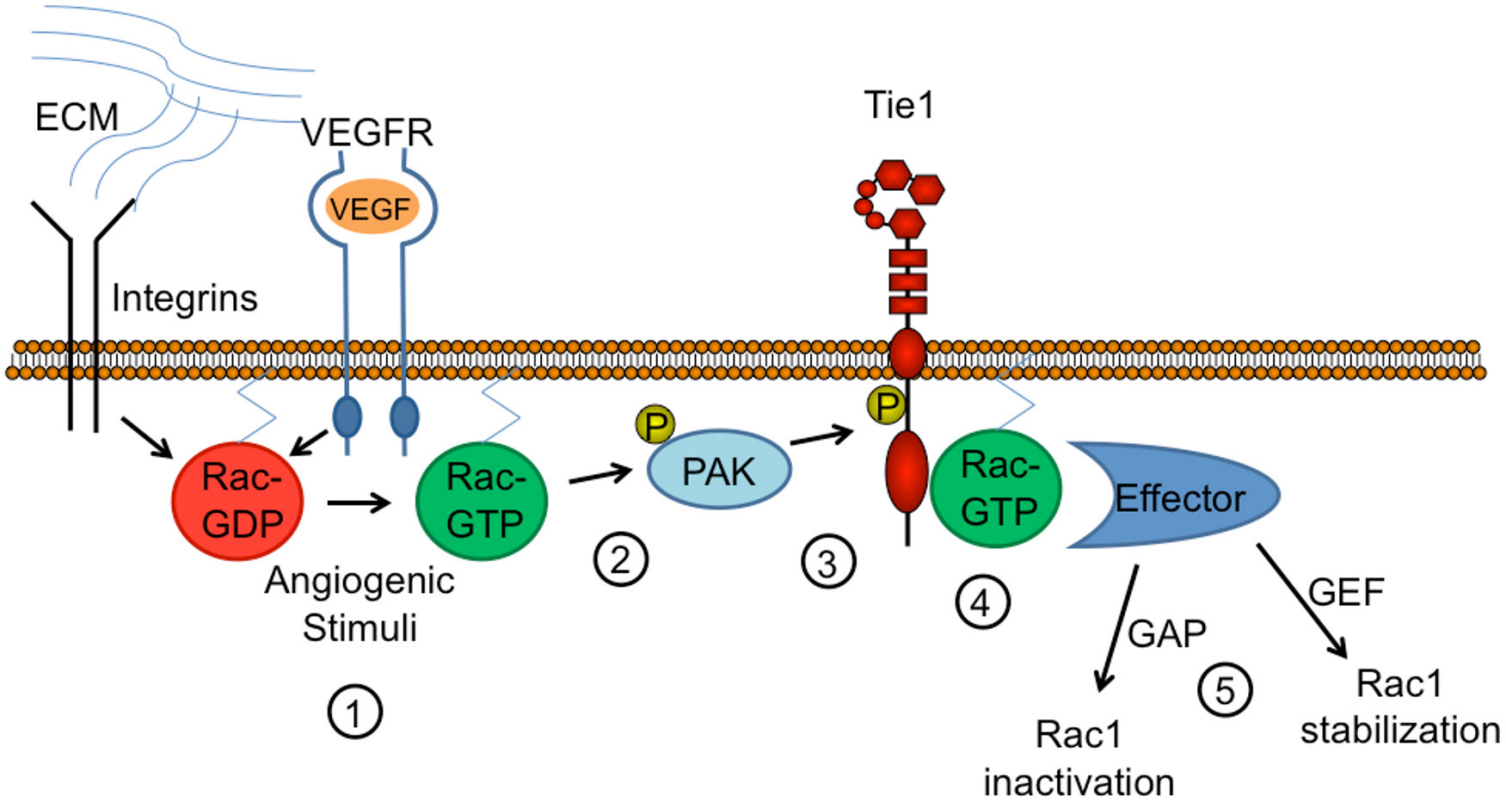
Proposed model for Tie1-T794 phosphorylation by Rac1/PAK1 and modulation of Rac1 signaling. **1)** Angiogenic stimuli (e.g., extracellular matrix (ECM)-mediated integrin activation, VEGF stimulation, etc.) induce Rac1 activation (Rac1-GTP). **2)** Rac1-GTP activates PAK1, resulting in PAK1 autophosphorylation. **3)** PAK1 phosphorylates Tie1 on T794. **4)** Rac1 associates with Tie1-pT794. **5)** Rac1 association with Tie1 facilitates interaction with effector molecules that either stabilize it in its active GTP-bound form or mediate GTP hydrolysis and subsequent Rac1 inactivation. Mutation of Tie1-T794 therefore disrupts Rac1 signaling and inhibits proper angiogenesis and capillary morphogenesis.

The finding that T794 phosphorylation occurs downstream of Rac1 activation, considered together with the fact that both Rac1 and Tie1-T794 are required for angiogenesis, suggests the presence of a feedback loop involving Rac1 activation, PAK1-mediated Tie1 phosphorylation, Rac1-Tie1 association, and subsequent modulation of angiogenesis (Fig 6). Whether this interaction serves to enhance or limit Rac1 activity remains unclear. Although further studies are needed to fully understand the nature of the relationship between Tie1-pT794, PAK1, and Rac1, this novel phosphorylation event and interaction provide important new insights into a previously unexplored role for Tie1 in the regulation of angiogenesis and capillary morphogenesis.

## Supporting Information

S1 ARRIVE Checklist NC3Rs ARRIVE Guidelines Checklist. Completed ARRIVE Guidelines checklist.
(DOCX)

S1 Dataset Compressed Proteomics Dataset. ZIP file containing raw data from Tie1 proteomic interactions following overexpression of Tie1-WT or -T794A in endothelial cells infected with or without AdAkt.
(ZIP)

S1 Fig T794A mutation inhibits endothelial cell migration. HUVECs were infected for 24h with adenoviruses encoding the indicated proteins then plated in 8μm Transwell filters coated with gelatin (0.1%) and allowed to migrate for 6h. Migrated cells were then fixed in methanol, stainted with DAPI and nuclei of migrated cells were then imaged by epiflourescence microscopy. Cells were counted in 3 random fields (4x magnification) using Image J. Data represents relative means ± SEM. * GFP vs TA, P<0.001.
(TIF)

S2 Fig T794 mutation does not affect Ang1-mediated tyrosine phosphorylation of Tie1 or downstream signaling. HUVECs were left uninfected or infected with AdTie1-WT or -T794A for 24 hours, starved in serum-free medium for 3 hours, then stimulated for 15 minutes with Ang1 (500ng/ml). Tie1 was immunoprecipitated (IP) from cell lysates and western blotted as indicated. Tie1-WT and T794A bands are from the same blot and exposure.
(TIF)

S3 Fig T794 phosphorylation state does not alter tyrosine phosphorylation of Tie2 or downstream signaling. HUVECs were uninfected or infected with AdTie1-WT or -T794A for 24 hours, starved in serum-free medium for 3 hours, then stimulated for 15 minutes with Ang1 (500ng/ml). Tie2 was immunoprecipitated (IP) from cell lysates and western blotted as indicated.
(TIF)

S4 Fig Tie1-mediated increase in inflammatory adhesion is independent of T794 phosphorylation. Confluent HUVECs were infected for 24h with the indicated proteins and then treated with or without TNFα (1ng/ml) overnight. U937 leukocytes (3x10^6^) were then incubated with the HUVECs for 30 min at 37°C, and non-adhered cells were washed away. Brightfield microscopy images were taken and the number of adherent U937 leukocytes was counted in 3 random 4x fields. Tie1 overexpression enhanced the inflammatory response to TNFα but in a T794-independent manner. Results are shown as means ± SEM. *, P<0.04.
(TIF)

S5 Fig Endothelial cell survival and proliferation are unaffected by mutation of T794. **a.** Uninfected or Adeno-infected HUVECs were left untreated or starved for 48 hours. Cells were fixed, stained with hematoxylin, and counted. Cell counts were normalized to the average number of cells per field in the untreated group for each virus group. b. HUVECs were infected for 24h with adenoviruses encoding the indicated proteins, and then sparsely plated 6-well plates. Separate groups of cells were fixed, stained, and counted every 24h after plating. Results are expressed as relative mean cell number ± SEM. Overexpression of Tie1 suppressed proliferation in a T794-independent manner. *, P<0.001.
(TIF)

S6 Fig Various RTK ligands fail to induce detectable T794 phosphorylation. HUVECs adenovirally overexpressing Tie1-WT were starved for 3h and stimulated for 15 minutes at 37°C with the following ligands: Ang1 (500ng/ml), Ang2 (500ng/ml), VEGF (25ng/ml). Tie1 was immunoprecipitated from whole cell lysates and both were western blotted as indicated. Bands from the AdmyrAkt-treated cells were from the same blot and exposure.
(TIF)

S1 Table Proteomic Tie1 interactions. Tie1 antibody was conjugated to beads and used to immunoprecipitate (IP) adenovirally overexpressed Tie1-WT or -T794A from endothelial cells that had also been infected with or without AdAkt. IPs were thoroughly washed and submitted to the Duke Proteomic core facility for mass spectrometric (LC-MS/MS) analysis.
(TIF)
