# Supplementary material for: Phosphorylation of Threonine 794 on Tie1 by Rac1/PAK1 Reveals a Novel Angiogenesis Regulatory Pathway
Source: PLoS One. 2015 Oct 5;10(10):e0139614. doi: 10.1371/journal.pone.0139614 (PMC4593579; doi:10.1371/journal.pone.0139614)

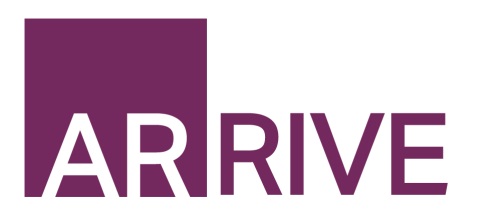


The ARRIVE Guidelines Checklist

Animal Research: Reporting In Vivo Experiments

Carol Kilkenny^1^, William J Browne^2^, Innes C Cuthill^3^, Michael Emerson^4^ and Douglas G Altman^5^

*^1^The National Centre for the Replacement, Refinement and Reduction of Animals in Research, London, UK, ^2^School of Veterinary Science, University of Bristol, Bristol, UK, ^3^School of Biological Sciences, University of Bristol, Bristol, UK, ^4^National Heart and Lung Institute, Imperial College London, UK, ^5^Centre for Statistics in Medicine, University of Oxford, Oxford, UK.*

|  | | ITEM | RECOMMENDATION | Section/ Paragraph |
| --- | --- | --- | --- | --- |
| 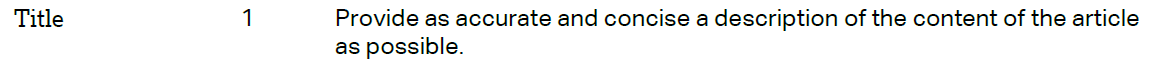 | | | Title page, lines 1-2 |  |
| 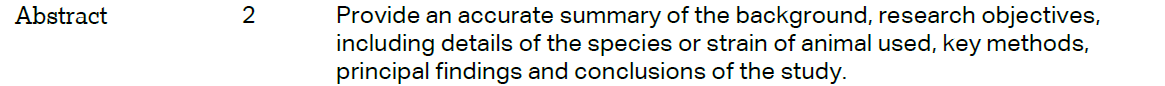 | | | Page 2, Abstract |  |
| INTRODUCTION | | |  |  |
| 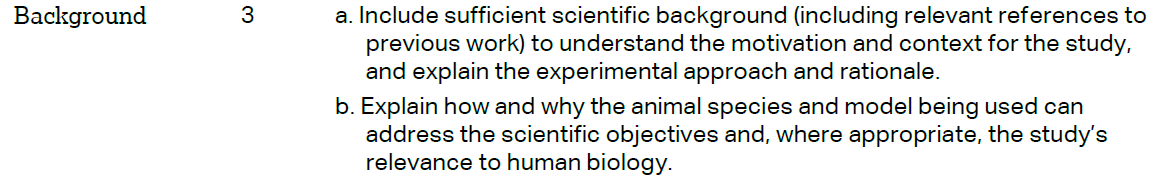 | | | Paragraphs 1-4 |  |
| 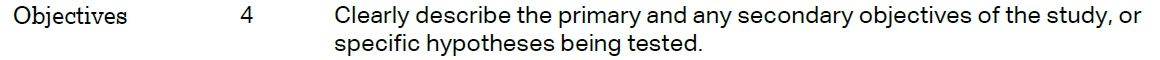 | | | Paragraphs 3&4 |  |
| METHODS | | |  |  |
| 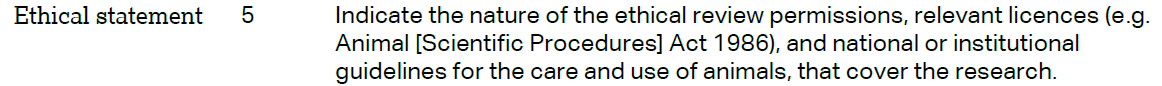 | | | Lines 122-123 |  |
| 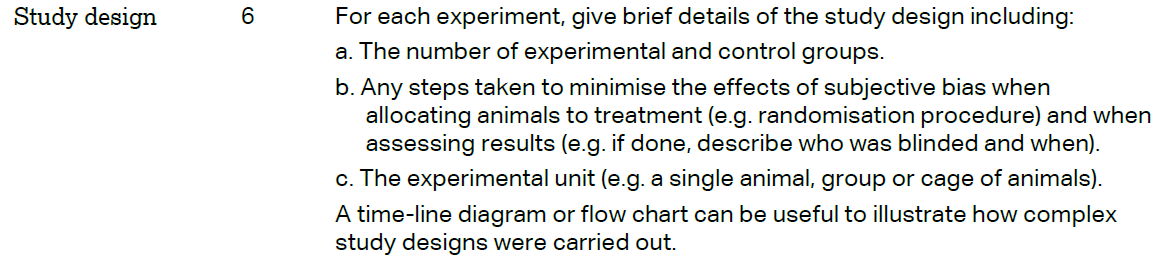 | | | Lines 129-140 |  |
| 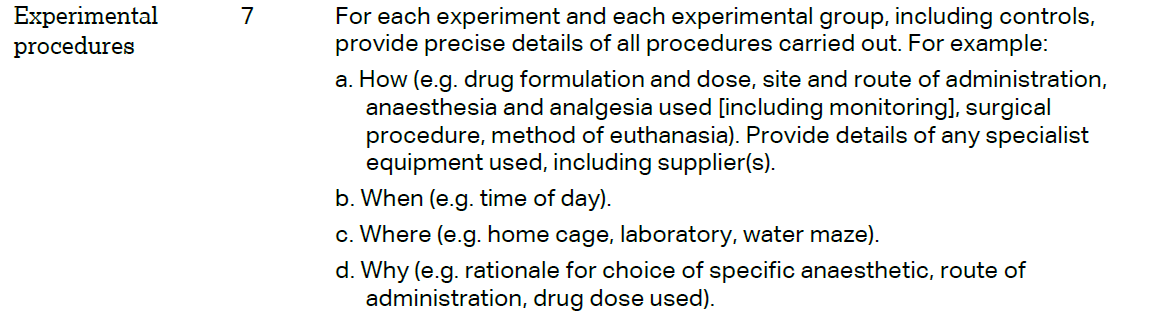 | | | Lines 123-140 |  |
| 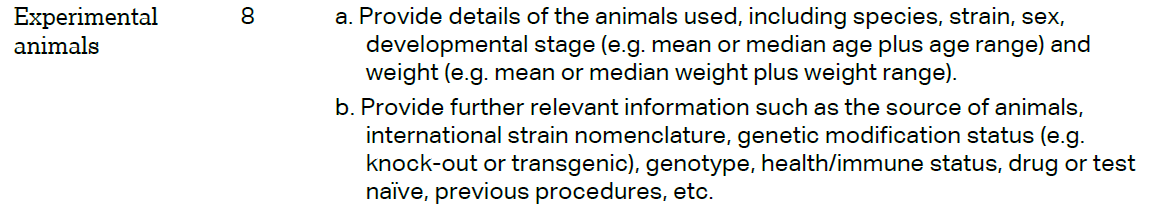 | | | Line 123-130 |  |

The ARRIVE guidelines. Originally published in *PLoS Biology*, June 2010^1^

| 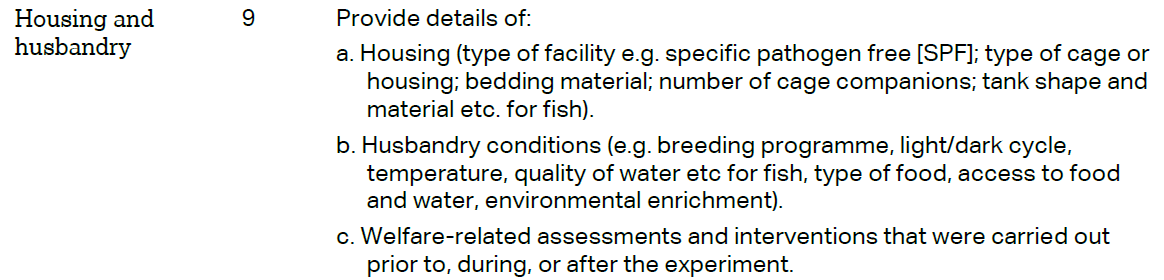 | Reference 18, lines 124-126 | |
| --- | --- | --- |
| 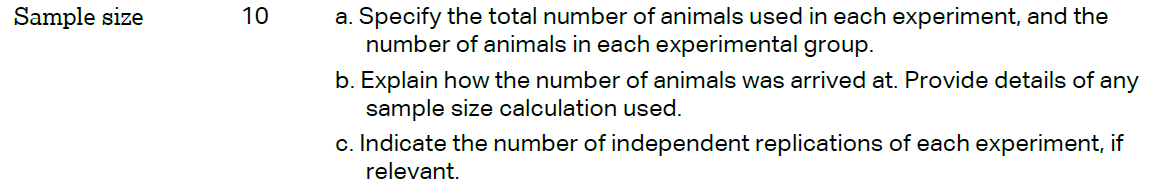 | Lines 138-140 | |
| 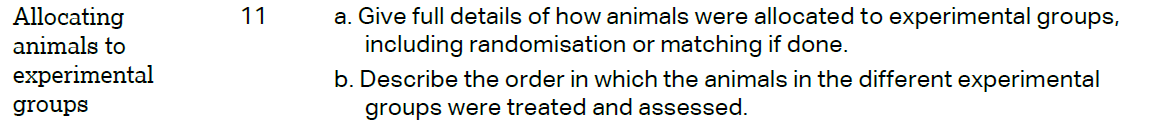 | Lines 130-133 | |
| 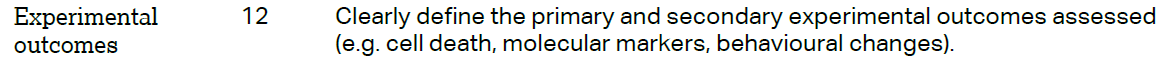 | Lines 133-135 | |
| 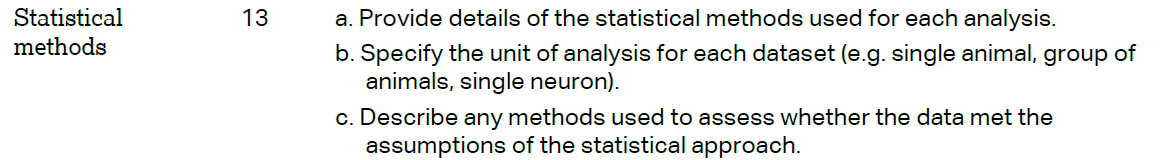 | Lines 212-214 | |
| RESULTS |  | |
| 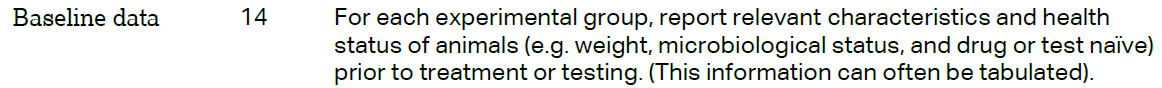 | Line 252-253 | |
| 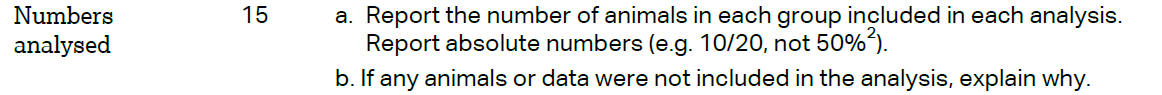 | Lines 258-274 | |
| 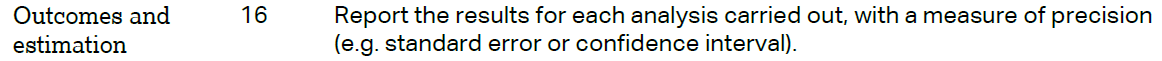 | Figure legends | |
| 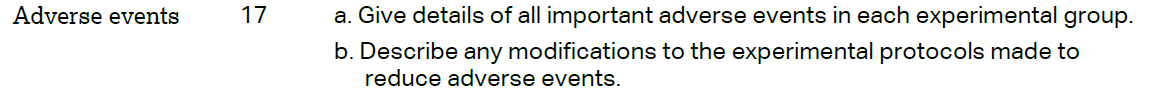 | N/A | |
| DISCUSSION |  | |
| 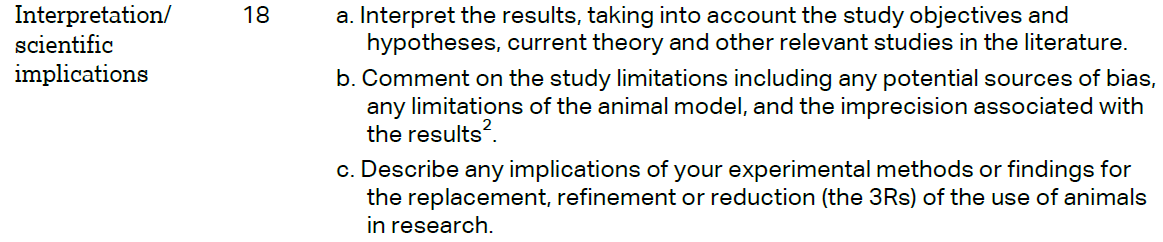 | Lines 404-434 | |
| 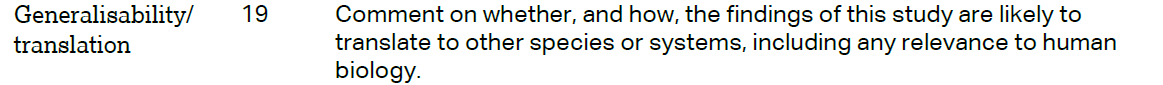 | Lines 488-503 | |
| 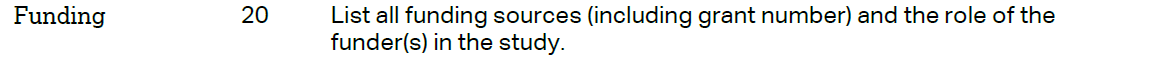 | | Online funding statement |


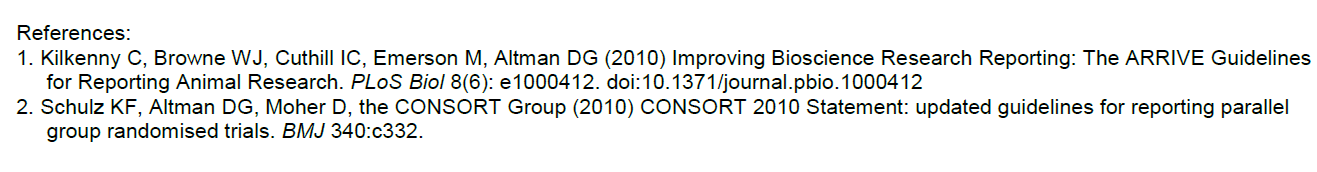

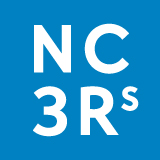

Supplement: S1 ARRIVE Checklist — Completed ARRIVE Guidelines checklist. (DOCX) [file pone.0139614.s001.docx]
