# Supplementary figures and images for: Phosphorylation of Threonine 794 on Tie1 by Rac1/PAK1 Reveals a Novel Angiogenesis Regulatory Pathway
Source: PLoS One. 2015 Oct 5;10(10):e0139614. doi: 10.1371/journal.pone.0139614 (PMC4593579; doi:10.1371/journal.pone.0139614)

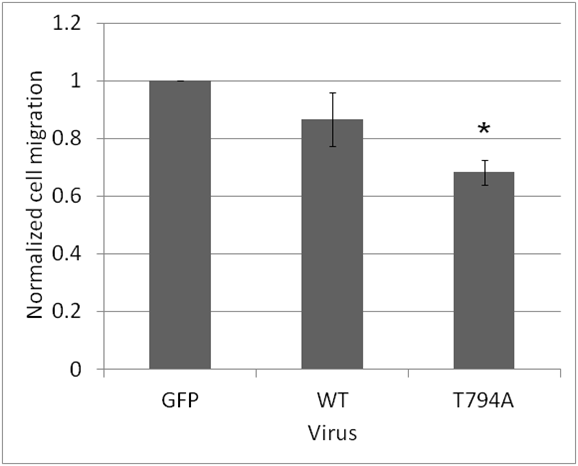

Supplement: S1 Fig — HUVECs were infected for 24h with adenoviruses encoding the indicated proteins then plated in 8μm Transwell filters coated with gelatin (0.1%) and allowed to migrate for 6h. Migrated cells were then fixed in methanol, stainted with DAPI and nuclei of migrated cells were then imaged by epiflourescence microscopy. Cells were counted in 3 random fields (4x magnification) using Image J. Data represents relative means ± SEM. * GFP vs TA, P<0.001. (TIF) [file pone.0139614.s003.tif]

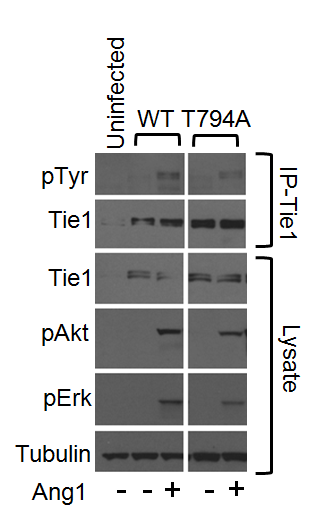

Supplement: S2 Fig — HUVECs were left uninfected or infected with AdTie1-WT or -T794A for 24 hours, starved in serum-free medium for 3 hours, then stimulated for 15 minutes with Ang1 (500ng/ml). Tie1 was immunoprecipitated (IP) from cell lysates and western blotted as indicated. Tie1-WT and T794A bands are from the same blot and exposure. (TIF) [file pone.0139614.s004.tif]

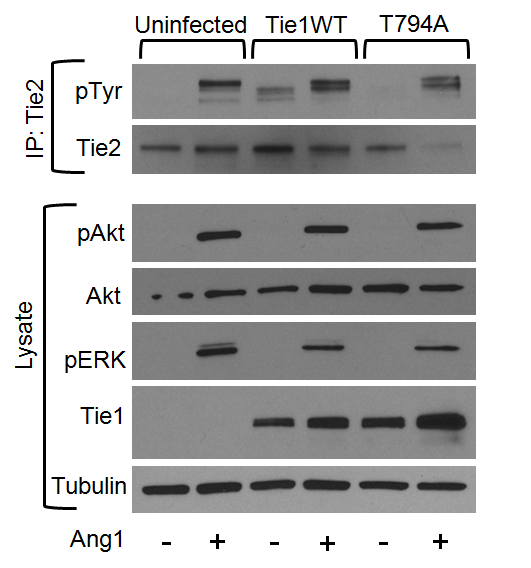

Supplement: S3 Fig — HUVECs were uninfected or infected with AdTie1-WT or -T794A for 24 hours, starved in serum-free medium for 3 hours, then stimulated for 15 minutes with Ang1 (500ng/ml). Tie2 was immunoprecipitated (IP) from cell lysates and western blotted as indicated. (TIF) [file pone.0139614.s005.tif]

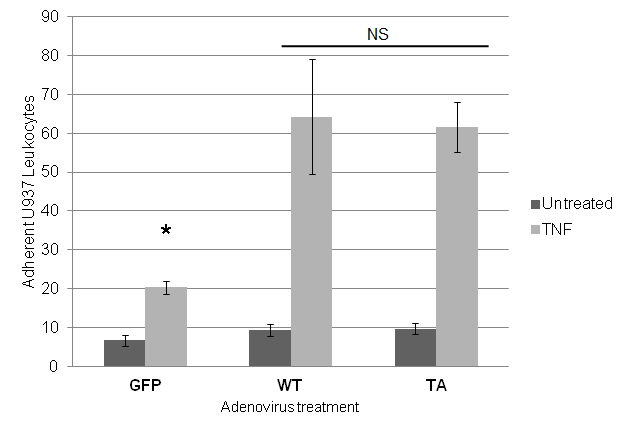

Supplement: S4 Fig — Confluent HUVECs were infected for 24h with the indicated proteins and then treated with or without TNFα (1ng/ml) overnight. U937 leukocytes (3x10^6^) were then incubated with the HUVECs for 30 min at 37°C, and non-adhered cells were washed away. Brightfield microscopy images were taken and the number of adherent U937 leukocytes was counted in 3 random 4x fields. Tie1 overexpression enhanced the inflammatory response to TNFα but in a T794-independent manner. Results are shown as means ± SEM. *, P<0.04. (TIF) [file pone.0139614.s006.tif]

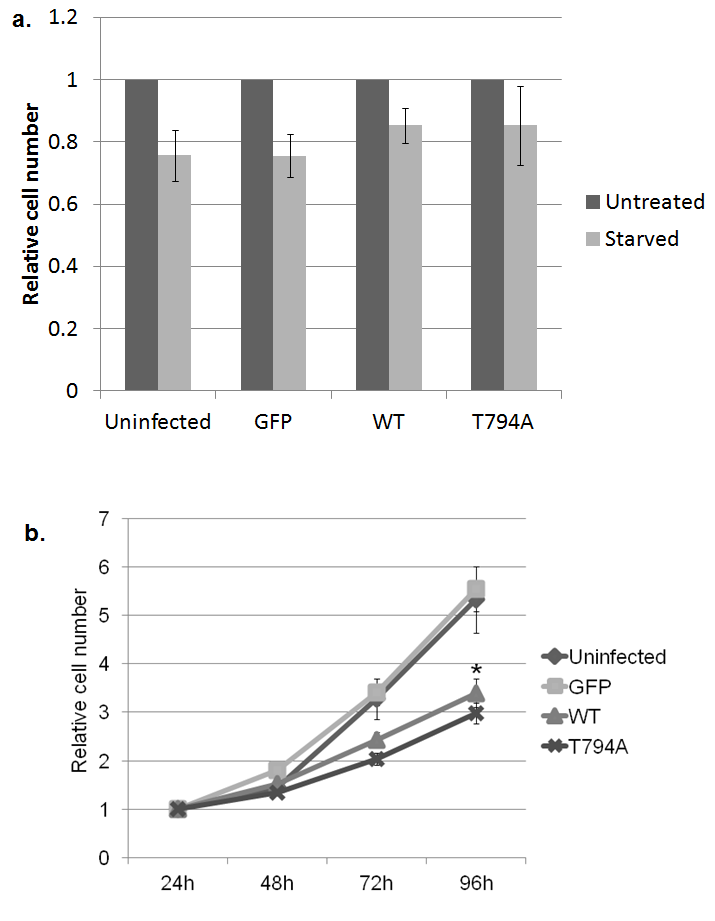

Supplement: S5 Fig — a. Uninfected or Adeno-infected HUVECs were left untreated or starved for 48 hours. Cells were fixed, stained with hematoxylin, and counted. Cell counts were normalized to the average number of cells per field in the untreated group for each virus group. b. HUVECs were infected for 24h with adenoviruses encoding the indicated proteins, and then sparsely plated 6-well plates. Separate groups of cells were fixed, stained, and counted every 24h after plating. Results are expressed as relative mean cell number ± SEM. Overexpression of Tie1 suppressed proliferation in a T794-independent manner. *, P<0.001. (TIF) [file pone.0139614.s007.tif]

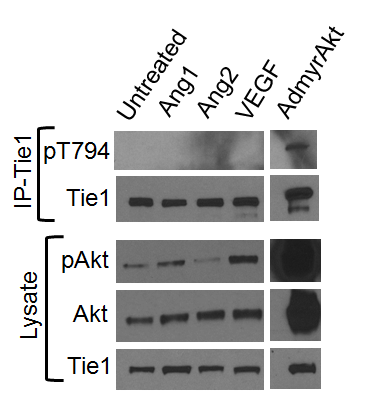

Supplement: S6 Fig — HUVECs adenovirally overexpressing Tie1-WT were starved for 3h and stimulated for 15 minutes at 37°C with the following ligands: Ang1 (500ng/ml), Ang2 (500ng/ml), VEGF (25ng/ml). Tie1 was immunoprecipitated from whole cell lysates and both were western blotted as indicated. Bands from the AdmyrAkt-treated cells were from the same blot and exposure. (TIF) [file pone.0139614.s008.tif]

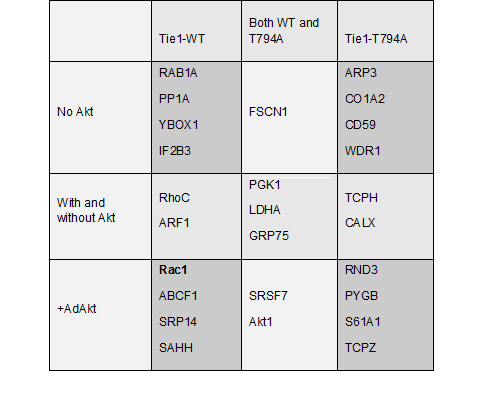

Supplement: S1 Table — Tie1 antibody was conjugated to beads and used to immunoprecipitate (IP) adenovirally overexpressed Tie1-WT or -T794A from endothelial cells that had also been infected with or without AdAkt. IPs were thoroughly washed and submitted to the Duke Proteomic core facility for mass spectrometric (LC-MS/MS) analysis. (TIF) [file pone.0139614.s009.tif]
